# The association between peer‐victimisation and structural and functional brain outcomes: A systematic review

**DOI:** 10.1002/jcv2.12081

**Published:** 2022-05-12

**Authors:** Tianyuan Ke, Sara De Simoni, Edward Barker, Patrick Smith

**Affiliations:** ^1^ Developmental Psychopathology Lab King's College London Institute of Psychiatry, Psychology & Neuroscience London UK; ^2^ Department of Psychology Institute of Psychiatry, Psychology and Neuroscience King's College London London UK

**Keywords:** adolescents, bullying, children, cyberbullying, emerging adults, neuroimaging, peer‐victimization, psychopathology

## Abstract

**Background:**

Peer adversity and aggression are common experiences in childhood and adolescence which lead to poor mental health outcomes. To date, there has been no review conducted on the neurobiological changes associated with relational peer‐victimisation, bullying and cyberbullying.

**Methods:**

This systematic review assessed structural and functional brain changes associated with peer‐victimisation, bullying, and cyberbullying from 1 January 2000 to April 2021. A systematic search of Psychoinfo, Pubmed, and Scopus was performed independently by two reviewers using predefined criteria. Twenty‐six studies met the selection criteria and were considered for review.

**Results:**

The data collected shows altered brain activation of regions implicated in processing reward, social pain, and affect; and heightened sensitivity and more widespread activation of brain regions during acute social exclusion, most notably in the amygdala, left parahippocampal gyrus, and fusiform gyrus, associated with victimisation exposure. In addition, victimised youths also demonstrated greater risk‐taking behaviours following acute social exclusion showing greater ventral striatum—inferior frontal gyrus coupling, activation in the bilateral amygdala, orbital frontal cortex (OFC), medial prefrontal cortex (MPFC), temporoparietal junction (TPJ), medial posterior parietal cortex (MPPC) and dorsomedial prefrontal cortex (dmPFC), suggesting greater social monitoring, seeking of inclusion, and more effortful cognitive control. The studies included participants from a very broad developmental age range, mostly using cross‐sectional measure of peer‐victimisation exposure, at varying developmental stages.

**Conclusions:**

This review highlights the need for more neuroimaging studies in cyberbullying, as well as longitudinal studies across more diverse samples for investigating gender, age, and developmental interactions with peer‐victimising. This also brings to attention the importance of addressing bullying victimisation particularly in adolescence, given the evidence for social stress in heightening developmentally sensitive processes which are associated with depression, anxiety, and externalising symptoms.

**Key points**

- Relational peer‐victimisation and bullying are associated with altered brain activation of regions implicated in processing reward, social pain, and affect.
- Heightened sensitivity, more widespread activation of brain regions during acute social exclusion.
- Victimised youths demonstrate greater risk‐taking behaviours following acute social exclusion.
- Existing gap in neuroimaging studies in cyberbullying.
- Social stress in adolescence heightens developmentally sensitive processes and is associated with psychopathology symptoms.

## INTRODUCTION

Bullying and cyberbullying are common in adolescence, with approximately a 25% lifetime prevalence for traditional bullying‐victimisation and 7% for cyber bullying victimisation (Jadambaa et al., 2019). Bullying‐victimisation leads to depression and anxiety symptoms within a year of exposure (Schoeler et al., 2018), but both bullying and cyberbullying have been associated with poor mental health outcomes (Baier et al., 2019). This is particularly striking during adolescence, a period of development of social interaction, identity and brain changes, and increased risk of psychopathology. Bullying is the general term used to describe the form of abuse between peers, defined as the repeated and chronic exposure to negative actions from one or more other people, including physical contact, words, gestures, and intentional exclusion. This results in an asymmetric relationship with an imbalance of power, and the victim having difficulties defending themselves (Olweus, 1993, 2010). Bullying may take both physical and non‐physical forms, either overt physical, verbal attacks and discrimination, or relational victimisation, including exclusion and ostracism by peers.

Similarly, cyberbullying includes the characteristics of traditional bullying but may be more difficult to define based on the criteria of intention to cause harm, repetition of attempts, or the imbalance of power, in the cyber context (Corcoran et al., 2015). In addition, there appear to be some differences in the predictors of increased risk of being bullied traditionally and online, with loneliness and school experience being more related to traditional bullying, while self‐esteem, family belonging, and parental involvement are associated with cyberbullying (Brighi et al., 2012). However, adolescents experience heightened social experiences of peer acceptance and rejection on social media platforms than they would in face‐to‐face interactions (Crone & Konijn, 2018), suggesting increased sensitivity to cyber peer abuse.

While there has been a strong interest in comparing cyberbullying and traditional bullying (Camerini et al., 2020), the effects of both types of peer‐victimisation on social cognitive‐affective related functional and structural brain differences, are not well understood. Given the chronic and repetitive nature of bullying, peer abuse contribute to an adverse social environment, particularly during adolescence, which may amplify genetic and predisposed risks (Blakemore, 2012). Adolescence is a period of heightened sensitivity to valuation of social rewards (Blakemore & Mills, 2014). The current evidence has shown that adolescents exhibit activation in brain regions similar to adults when processing rewards, which are the ventral and dorsal striatum, posterior cingulate cortex, nucleus accumbens (NAcc), and the orbital frontal cortex (OFC), or the ventromedial prefrontal cortex (vmPFC) (Van Leijenhorst et al., 2010). Adolescents in comparison to adults, however, display greater likelihood of activation of the reward network when processing positively‐valenced stimuli, greater likelihood of activation in the insula during anticipation of reward, and greater activation in the amygdala and putamen when processing reward outcomes (Silverman et al., 2015).

Existing literature has also shown that risk‐taking is more prevalent in adolescence, particularly in the presence of peers (Gardner & Steinberg, 2005). This has been attributed to the combination of increased sensitivity to reward in the vmPFC, which peaks in adolescence, coupled with brain areas involved in cognitive control processes including the dorsal anterior cingulate cortex (ACC) and lateral prefrontal cortex (PFC), which show linear increases in activation with age (Van Leijenhorst et al., 2010). Greater activation has also been found in the VS and OFC, which predicted risk taking behaviours in the presence of peers during a driving simulation task (Chein et al., 2011). In the context of peer and social interaction, this results in greater response to emotionally appetitive stimuli (e.g., happy faces) reflected in the exaggerated ventral and dorsal striatum activation, while inferior frontal gyrus (IFG) activity reflects cognitive control demands during successful inhibition of prepotent responses (Somerville et al., 2011). Hence, emotion processing, which involves perceiving and recognising emotional stimuli, recruiting the affective (limbic) network (RajMohan & Mohandas, 2007), as well as brain regions for detecting threat and emotional salience, such as the amygdala, hippocampus, ACC, and PFC may also be important to understanding the impact of bullying and peer‐victimisation on the developing brain.

Furthermore, brain regions used to exert cognitive control enabling goal‐directed behaviours recruited in the process of modulating one's emotion (Braunstein et al., 2017) may also interact with peer‐victimisation exposure. The dorsolateral PFC (dlPFC) and ventrolateral PFC (vlPFC) facilitate the downregulation of negative affective responses experienced during social exclusion (Zhao et al., 2021). These regions function to allow for effective goal‐directed behaviours through holding in mind information and strategies, whilst inhibiting conflicting responses (Baird et al., 2010). The dlPFC, vlPFC, and their coupling with other key regions such as the amygdala, develop through adolescence and childhood and are associated with more successful down‐regulation of negative affect with age (Silvers et al., 2017).

There are gaps in the existing literature that can be addressed, which include elucidating the impact of chronic and repetitive peer social stressors on the developing brain, and their contribution to increased risk for poor mental health outcome. This review seeks to address this gap and uncover any potential differences in the impact of different forms of bullying, including cyberbullying and cyber‐victimisation, on structural and functional brain differences. The aim of this review is to address the following: (1) are structural and functional brain differences associated with bullying?; (2) are structural and functional brain differences associated with cyberbullying?; and (3) do structural and functional brain differences differ between traditional bullying and cyberbullying?

To our knowledge, no recent reviews have been conducted specifically on the neurobiological differences associated with bullying and cyberbullying. An existing review summarised the effects of bullying experience using neuroscience, neuroendocrinology and genetics evidence (Vaillancourt et al., 2013), although only a few neuroscience studies were included to demonstrate the similarity between social pain and physical pain. A recent review on bullying‐victimisation in childhood and adolescence did not find strong evidence for a causal relationship between bullying‐victimisation and adverse psychosocial problems (Moore et al., 2017). However, given that neuroimaging studies have become more prevalent in the last 10 years, there is potential novelty to undertaking this review to cover the recent developments in neuroimaging evidence of bullying on brain structure and function, potentially providing more evidence for the causality of bullying in association with poor mental health outcomes.

## METHODS

### Protocol and registration

This review protocol was registered in the National Institute for Health Research's International prospective register of systematic review PROSPERO on 30/09/2020 (registration number CRD42020186476).

### Inclusion and exclusion criteria

Studies were included if they (1) involved participants who were children, adolescents, and young adults up to 25 years old, (2) were published in the last 20 years (since 1 January 2000), (3) included measures of exposure to peer victimisation, bullying, or cyberbullying by self‐ or other‐report, which is associated with relevant outcomes and (4) compared individuals with high exposure to peer‐victimisation, bullying, or cyberbullying to control participants with low exposure to peer‐victimisation, bullying, or cyberbullying; or reported the correlations between exposure to peer victimisation, bullying, or cyberbullying and relevant outcomes, and (5) reported structural and functional neuroimaging findings with the use of MRI techniques including diffusion tensor imaging (DTI) to assess structural connectivity, task‐based and resting‐state functional MRI (fMRI) or electroencephalogram (EEG) to assess brain activity and functional connectivity. There was no minimum sample size for inclusion in the review.

### Identification of studies

Studies were identified through searches of electronic databases (Psychinfo, Pubmed, and Scopus). The search strategy is reported in the Supporting Information. In addition, a manual search was conducted of the reference lists of selected papers and of relevant review articles retrieved by the database. Grey literature search of conference papers and doctoral theses was conducted using the Bielefeld Academic Search Engine (BASE).

### Study selection

**FIGURE 1:**
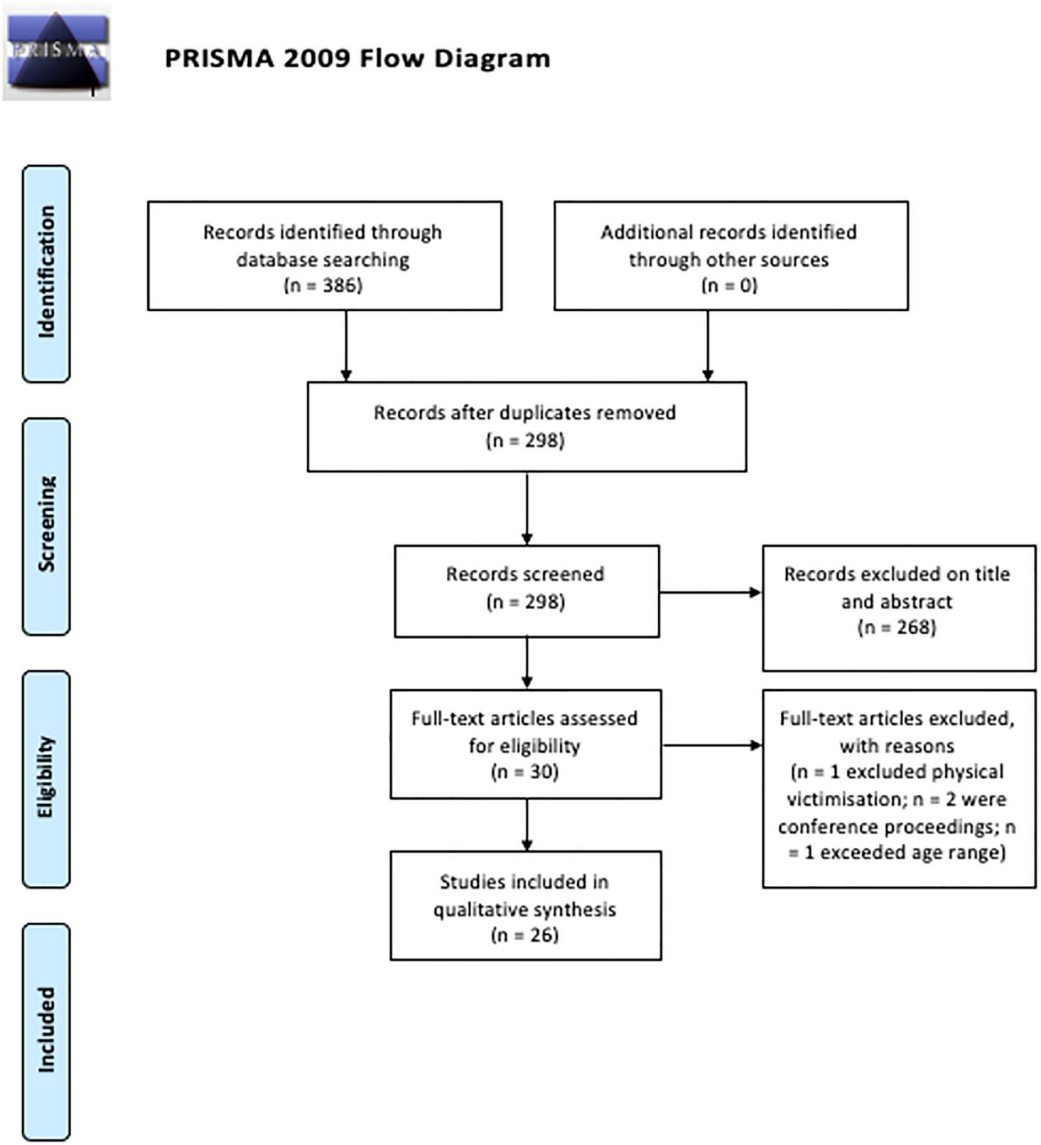
PRISMA flow chart describing study screening and inclusion process

The PRISMA flowchart was used to report inclusion of studies (Figure 1). First, all the results were screened by title and abstracts, independently by two reviewers. Any discrepancies were resolved through discussion and further screening of the article full texts until consensus was achieved. Applying the inclusion and exclusion criteria, studies were excluded for the following reasons: ongoing studies reported as conference presentations; conference presentations not presented as peer‐reviewed journal articles; study that excluded specific forms of peer‐victimisation (i.e., peer physical bullying) as well as poor study quality rating, and age of sample.

### Data extraction

The following details were extracted from each study: study design, populations, use of control groups if any, sample size, age, measure of exposure, experimental task/manipulation, neuroimaging modality and analysis, processing pipeline, region(s) of interest, and findings (Table 1).

**TABLE 1:** Summary of included studies

Author	Year	Study population	Control/comparison group	*N*	Age	Exposure measure (s)	Task (contrast)	Software/pipeline	Neuroimaging method	ROIs/seed(s)/constraints	Findings
Baird et al.	2010	Female adolescents	NA	14	*M*_age_ = 14.16 (Range = 12.8–15.2)	Authors developed a new self‐report measure of relational aggression	Affect recognition of happy, sad, afraid, angry, and neutral face (face, fixation cross)	SPM99	tb‐fMRI	Anterior cingulate, bilateral middle frontal gyrus, dlPFC, R posterior cingulate, fusiform gyrus, R middle frontal gyrus, L superior frontal gyrus, amygdala	Lower relational aggression predicted increased signal intensities in response to female peer faces (vs. fixation) in the posterior cingulate, anterior cingulate, R middle frontal gyrus and L middle frontal gyrus (amygdala was non‐significant)
Casement et al.	2014	Female young adolescents	NA	120	16	Peer Experiences Scale I (Vernberg et al., 1999) at age 11–12	Monetary reward guessing task (Forbes et al., 2009, 2010)	SPM8, PickArlas 3.0.3	tb‐fMRI	Striatum, mPFC, orbitofrontal cortex (OFC), and amygdala	Peer‐victimisation was associated with decreased response to potential rewards in the mPFC
du Plessis et al.	2019	Children	NA	50	*M*_age_ = 9.29; 14 at follow‐up	Olweus Bully/Victim questionnaire (Olweus, 1986) self‐report of bullying and victimisation	NA	Freesurfer 6.0 (segmented subcortical volumes)	sMRI	NA	vlPFC surface area, GMV, and thickness 5 years later dependent on cortisol levels and diurnal slope, but only in boys
Eckstrand et al.	2019	Adolescents of sexual minority, non‐clinical	NA	46	14–18	Retrospective report of neighbourhood and school crime, peer victimisation, and discrimination from ages 10–16	Social reward task (inclusion, exclusion), monetary reward task (gain, loss)	SPM8	tb‐fMRI	WBA determined ROIs—R mPFC, L AI, R TPJ	Non‐significant effect of victimisation
Ethridge et al.	2018	Emerging adults	NA	61	*M*_age_ = 20.20 (SD = 1.51; Range = 18–25)	Peer Experiences Scale (Vernberg et al., 1999) measure of past year relational (social exclusion) and physical victimisation	Doors task	32‐channel EEG	EEG/ERP	Reward positivity (RewP) to doors task monetary reward	Past‐year relational, but not physical, victimisation was associated with a blunted neural response to rewards
Fowler et al.	2021	Study 1: Adolescents (community); Study 2: Female adolescents	Study 1: NA; Study 2: Low victimisation (*n* = 9), chronic victimisation (*n* = 17)	Study 1: 33; Study 2: 26	Study 1: *M*_age_ = 13.71 (SD = 2.71); study 2 (*M*_age_ = 15.43, SD = 0.32)	Social Experiences Questionnaire‐ Revised (SEQ‐R; Rudolph et al., 2014) 12‐item self‐report of relational, overt and cyber peer victimisation	Social evaluation task	SPM8	tb‐fMRI & PPI	Study 1: WBA when perceiving low > high relational value; Study 2: ventral striatum (VS) as seed region	Study 1: peer‐victimisation associated with reduced FxC between VS and bilateral IFG, R putamen, and greater FxC between VS‐L inferior occipital gyrus, when perceiving low relative to high relational value
											Study 2: Peer‐victimisation was associated with reduced FxC between VS‐bilateral IFG (but not mPFC) when perceiving low relative to high relational value
Jarcho et al.	2019	Adolescents	Low victimisation (*n* = 20), high victimisation (*n* = 27)	47	11 (at time point 1)	Kids in My Class (KIMC) questionnaire (Kochenderfer & Ladd, 1996)	Virtual School paradigm (peer evaluation)	AFNI	tb‐fMRI	Bilateral striatum (caudate, Nacc, and putamen), amygdala, dorsal ACC, and insula	In victimised participants, high wariness and right amygdala response to unpredictably positive peer evaluation was associated with more severe social anxiety symptoms
Lee et al.	2014	Children	Peer‐rejected (*n* = 14), control group (*n* = 10)	24	5–6th grade	A 12‐item self‐report Ostracism Scale developed by authors to assess experiences of peer rejection, based on a normative sample of 2972 middle school students	Receiving facial expressions feedback (positive, negative, neutral) following performance on unsolvable geometric puzzles	SPM8	tb‐fMRI	CD	Peer‐rejection group exhibited greater and more extensive activation of the amygdala, OFC and vlPFC in response to negative feedback stimuli of emotional faces
Lee et al.	2020	Adolescent girls (MDD)	MDD (*n* = 56), healthy controls (*n* = 47)	103	12–17	Peer‐Victimization Scale (PVS) & Bullying‐Behaviour Scale (BBS) summed score	NA	Freesurfer 6.0 (segmented subcortical volumes)	sMRI	Nacc, amygdala, hippocampus	Increased Nacc volume (but not the amygdala and hippocampal volumes) explained the indirect effect of peer problems on depression symptoms
McIver et al.	2019	Adolescents	peer victimized (*n* = 15), defenders (*n* = 15), and controls (*n* = 15)	45	17.7	Bully and Victimization Questionnaire (BVQ) is a self‐report measure of bullying being victimized by peers; Electronic Bullying Roles Questionnaire (EBRQ) self‐report of participation in prosocial, cyber‐defending behaviour	Cyberball (social inclusion, exclusion)	SPM12/CONN Toolbox	FxC	ACC, MPFC, R insula, L amygdala	Peer‐victimised group showed weaker ROI‐to‐ROI connectivity between the ACC, L amygdala, and R insula. Significant moderating effect for continuous FxC of the MPFC‐ L amygdala on the relationship between peer‐victimisation and depressive symptoms (collapsed across conditions)
McIver et al.	2018	Adolescents	peer victimized (*n* = 15), defenders (*n* = 15), controls (*n* = 15)	45	17.7	Bully and Victimization Questionnaire (BVQ) self‐report of peer bullying/victimisation; Electronic Bullying Roles Questionnaire (EBRQ) self‐report of prosocial, cyber‐defending behaviour	Cyberball (social inclusion, exclusion)	SPM12	tb‐fMRI	WBA derived ROIs based on victimisation between‐group: amygdala, parahippocampal gyrus, IFG, and fusiform gyrus	Peer‐victimisation was associated with increased neural response in the L amygdala, L parahippocampal gyrus, L inferior frontal operculum, and R fusiform gyrus
McLoughlin et al.	2020	Adolescents/emerging adults	cyberbullying, cybervictims, cyberbully‐victims	32	18–25	Berlin Cyberbullying‐Cybervictimization Questionnaire measure of cyberbullying and cybervictimization (Schultze‐Krumbholz & Scheithauer, 2009, 2011)	Cyberbullying Picture Series (CyPicS) (cyberbullying, neutral)	SPM12	tb‐fMRI	NA	Greater activation in L precuneus in subjects without prior experiences of cyberbullying compared to those with prior cyberbullying experiences when observing cyberbullying stimuli compared to neutral stimuli
Muetzel et al.	2019	Children	Perpetrator (*n* = 82), target (*n* = 92), combined perpetrator and target (*n* = 47)	221	10	Parent and teacher reported physical, verbal, and relational bullying	NA	Freesurfer 6.0 (segmented into WM, GM, CSF, and surface‐based WM/GM models)	sMRI	Precentral gyrus	Children classified as frequent targets of bullying showed thicker fusiform gyrus; non‐significant findings for hippocampus and amygdala
Oppeheimer et al.	2020	Adolescents (clinically anxious sample)	NA	36	11–16	Ecological momentary assessment of peer victimization; victim subscale of peer relations questionnaire	Chatroom interaction task (social acceptance and rejection feedback)	SPM12	fMRI	ROIs (a prior): bilateral AI, dACC; exploratory WBA	ROI: Youths reported higher levels of suicidal ideation if they exhibited heightened activation to social rejection in the R AI, and also experienced peer‐victimisation; WBA: increased activation in the R AI and inferior frontal gyrus, L inferior frontal gyrus, and R occipital gyrus associated with peer‐victimisation
Quinlan et al.	2018	Adolescents (community)	LCA high versus low peer victimisation	682	14–19 (baseline: 14.4 ± 0.4 (mean ± SD); follow‐up 1: 16.5 ± 0.6; follow‐up 2: 19.0 ± 0.7.)	Health Behaviour in School‐Aged Children (HSBC) measure of victimization during the previous 6 months	NA	SPM8	sMRI	Bilateral inferior OFC, ACC, insula, hippocampus, parahippocampal gyrus, amygdala, caudate, putamen, and thalamus	Peer‐victimisation was indirectly associated with generalized anxiety via decreases in putamen volume and L caudate ‐ individuals who have been chronically victimised have steeper decreases in putamen volume than their less victimised counterparts
Rappaport et al.	2019	Adolescents/emerging adults	NA	56	18.06 ± 1.01	Global Peer Relations scale of the Health and Behaviour Questionnaire (HBQ), measure of parent‐reported peer acceptance/rejection, physical victimization, and relational victimization (at ages 3–6; 15–18).	Island Gateway task (peer acceptance/rejection), Doors task (monetary reward/loss)	32‐channel EEG	EEG/ERP	Reward positivity (RewP) to peer acceptance and rejection feedback, money reward and loss feedback	Early and recent peer‐victimisation were associated with more blunted RewP, for social but not for monetary gains, after controlling for age and current depression symptoms
Rudolph et al.	2016	Female adolescents	NA	47	*M*_age_ = 15.46 (SD = 1.435)	Social Experiences Questionnaire a 21‐item revised version (for details, see Rudolph et al., 2014) of the Social Experiences Questionnaire (Crick & Grotpeter, 1996)	Cyberball (social inclusion, exclusion)	SPM8/AFNI	tb‐fMRI	WBA extracted ROIs associated with internalising symptoms dACC, sgACC and bilateral insula	dACC, sgACC, and insula activation were indirectly associated with internalising symptoms through avoidance in victimised but not non‐victimised girls
Rudolph et al.	2021	Female adolescents	NA	43	*M*_age_ = 15.44 (SD = 0.39)	Social Experiences Questionnaire revised version (Rudolph et al., 2014) based on the (Crick & Grotpeter, 1996) assessing overt victimisation, relational victimisation and cyber‐victimisation	Emotional faces matching task of negative (angry, fearful, sad) or positive (happy, surprised, calm) emotions (matching/viewing)	SPM8	fMRI/PPI	Bilateral amygdala	Victimisation significantly predicted more positive amygdala‐rVLPFC connectivity in girls with high but not low rejection sensitivity, during negative emotions condition (non‐significant for positive emotion condition)
Swartz et al.	2020	Adolescents (community)	NA	49	12–15	Relational aggression subscale of the Peer Experiences Scale (Prinstein et al., 2001) measure of experiences of relational peer aggression (both as the bully and the victim)	Emotional face matching of angry and fearful faces (geometric shapes)	SPM12	fMRI, exploratory WBA	Bilateral amygdala; exploratory WBA	Low amygdala activity to angry faces and fearful faces predicted lower levels of peer‐victimisation
Telzer et al.	2020	Female adolescents	NA	38	*M*_age_ = 15.43 (*SD* = 0.33)	Social Experiences Questionnaire—Revised (Rudolph et al., 2014), prospectively assessed across 2nd–8th grade	Social evaluation task	SPM8	tb‐fMRI	WBA	Greater exposure to peer‐victimisation was associated with heightened activation to in‐group relative to out‐group peers in the amygdala, ventral striatum, fusiform gyrus, and TPJ
Telzer et al.	2018	Female adolescents	chronically victimised (*n* = 25), non‐victimised (*n* = 21)	46	*M*_age_ = 15.3	Social Experiences Questionnaire—Revised (Rudolph et al., 2014), measure of overt and relational forms of peer victimization.	Stoplight risk‐taking task (risky/non‐risky decision), Cyberball (social exclusion/rejection)	SPM8	tb‐fMRI	WBA	Victimised girls showed greater reactivity in the bilateral amygdala, ventral striatum, and OFC, MPFC, TPJ, and MPPC, during risky decisions. Victimised girls also showed greater activation in the MPFC, DMPFC, TPJ, MPPC, STS, VLPFC and DLPFC when making ‘safe’ decisions (inhibiting risky response)
Vargas et al.	2019	Adolescents/emerging adults, clinically high risk	Clinical high‐risk (*n* = 41) and healthy volunteers (*n* = 48)	89	*M*_age_ = 17.57; 18.47	Autism‐Tics, ADHD, and other Comorbidities inventory (A‐TAC: Larson et al., 2013); Social Responsiveness Scale (SRS: Constantino et al., 2003) parent‐report items	NA	Freesurfer; TRActs Constrained by Under‐Lying Anatomy (TRACULA)	sMRI/DTI	ROIs; follow‐up exploratory WBA (hippocampus, amygdala, OFC, grey matter along with uncinate fasciculus white matter integrity)	Increased bullying exposure was associated with lower medial OFC volumes
Weissman et al.	2019	Adolescents	NA	133	17	Retrospective self‐report (ages 10–16) of neighbourhood and school crime, peer victimisation (physically, verbally, and emotionally), and discrimination based on ethnicity	Emotional face viewing (faces, fixation cross)	FSL, AFNI	fMRI	Coupling of mPFC‐ autonomic nervous system; vmPFC‐ respiratory sinus arrhythmia (RSA); dmPFC‐skin conductance response	Threat exposure was also associated with stronger negative coupling of the vmPFC‐RSA
Will, van Lier et al.	2016	Adolescents (community)	Chronically rejected (*n* = 19), stably high accepted (*n* = 27)	46	12–15	Children nomination of classmates they like and dislike	Cyberball (social inclusion, exclusion)	SPM8	tb‐fMRI	WBA	Chronically rejected adolescents showed higher activity in the dorsal anterior cingulate cortex and anterior prefrontal cortex during exclusion
Will, Crone et al.	2016	Adolescents	Stably accepted (*n* = 25), chronic peer‐rejected (*n* = 18)	43	14	Children nomination of classmates they like and dislike	Dictator game (money sharing/forgiveness or non‐sharing/retaliation) following Cyberball (social inclusion, exclusion)	SPM8	tb‐fMRI	WBA derived ROIs during punishment and forgiveness: bilateral ventral striatum, R dlPFC, bilateral parietal cortex, dmPFC	During forgiveness of excluders, chronically rejected adolescents showed enhanced activity in lPFC and dorsal striatum compared to stably rejected adolescents
Zhu, Lowen et al.	2019	Healthy young adults	NA	202	20–25	Maltreatment and Abuse Chronology of Exposure (MACE; Teicher & Parigger, 2015) scale measure of exposure to 10 types of maltreatment across each year of childhood	Emotional face‐matching of angry and fearful face (faces, geometrical shapes)	FEAT (FMRI Expert Analysis Tool) version 4.1.6, part of FSL	sMRI & tb‐fMRI	Bilateral amygdala	Peer emotional abuse during adolescence was associated with increased bilateral amygdala response to emotional faces versus shapes; peer physical abuse in early childhood was associated with decreased amygdala response to face versus shapes and decreased amygdala grey matter volume
								Freesurfer 6.0			

Abbreviations: ACC, anterior cingulate cortex; AFNI, Analysis of Functional Neuroimages; AI, anterior insula; CD, Cannot determine; dACC, dorsal anterior cingulate cortex; dmPFC, dorsomedial prefrontal cortex; DTI, Diffusion tensor imaging; EEG, electroencephalogram; ERP, event‐related potential; FxC, functional connectivity; MPFC, medial prefrontal cortex; MPPC, medial posterior parietal cortex; NA, Not applicable; NAcc, nucleus accumbens; OFC, orbitofrontal cortex; PPI, psychophysiological interaction; ROI, Region(s) of interest; sgACC, subgenual anterior cingulate cortex; sMRI, structural magnetic resonance imaging; SPM, Statistical Parametric Mapping; STS, superior temporal sulcus; tb‐fMRI, task‐based functional magnetic resonance imaging; TPJ, temporoparietal junction; vmPFC, ventromedial prefrontal cortex; WBA, whole brain analysis.

### Quality assessment

The NIH Quality Assessment Tool for Observational Cohort and Cross‐Sectional Studies was used to assess the quality of the included studies (Supporting Information). All included studies were rated by two independent reviewers and all discrepancies were resolved through discussion.

## RESULTS

### Description of studies and samples

A total of 26 studies were included in this review after screening, with only one study having a sole focus on cyberbullying. All included studies were rated as *fair* (*n* = 15) or *good* (*n* = 11) in quality. More information about study samples characteristics and exposure measures is presented in Table 1 below.

### Experimental manipulation

The neural response to social and peer evaluation in chronically victimised children and adolescents involves social acceptance and rejection processes or experiences of “social pain” previously described by Eisenberger et al. (2003), as well as reward processes, social and non‐social in nature. These social cognitive‐affective brain processes can be acutely brought on and investigated through a range of EEG and fMRI paradigms. Of the 26 included studies these, consisted of the widely used Cyberball (Davis et al., 2019; McIver et al., 2019, 2018; Will, Crone et al., 2016; Will, van Lier et al., 2016), Virtual School paradigm (Jarcho et al., 2019), Social Judgment task (Eckstrand et al., 2019), Peer evaluation task (Fowler et al., 2021; Jarcho et al., 2019; Telzer et al., 2020), Chatroom Interaction task (Oppenheimer et al., 2020), and the Island Gateway task (Rappaport et al., 2019; see Table 1). These paradigms involve a combination of the following manipulations: (1) making judgements on whether the research participant likes or dislikes peers, (2) receiving feedback about whether they are liked or disliked by others, and (3) being included or excluded in some form of social interaction with others.

### Neural substrates and processes implicated in peer victimisation

A range of psychological, behavioural, and neurobiological processes and their interactions with social or environmental factors were investigated by the papers included in this review. The findings will be summarised according to the various processes—social acceptance and rejection sensitivity, emotion processing and regulation, as well as social cognition and risk‐taking, to allow for synthesis and comparison of findings across different studies with minimal duplication of discussion.

#### Social acceptance and rejection

##### Reward sensitivity

The included studies found mixed evidence for peer‐victimisation associated with functional brain differences in the medial prefrontal cortex (mPFC) and VS, with decreased response observed for anticipation and response to monetary reward in adolescents and emerging adults (Casement et al., 2014). However, there were no signification association found between early peer‐victimisation and brain activation in the mPFC or VS to monetary reward in adolescents and emerging adults (Rappaport et al., 2019), nor chronic exposure to peer‐victimisation reported by sexual minority youths and activity in the right mPFC, left AI, and right temporoparietal junction (TPJ; Eckstrand et al., 2019).

Similarly, there were contradictory findings as to whether peer‐victimisation was associated with blunting of grand‐averaged reward‐related positivity (ReWP) to social acceptance in adolescents and emerging adults (Rappaport et al., 2019) or increased brain activation to social reward in the amygdala (Jarcho et al., 2019), VS, fusiform gyrus, and TPJ (Telzer et al., 2020), although this association was not reported in chronic exposure to peer‐victimisation reported by sexual minority youths and activity in the right mPFC, left AI, and right TPJ (Eckstrand et al., 2019).

There is evidence across the included studies that relational and overt peer‐victimisation, being *social* stressors, are differentially associated with reduced sensitivity to social and non‐social rewards, given the developmental timing of exposure. Evidence for altered activation within the reward network was found to be associated with early peer‐victimisation, general social stressors, as well as recent relational peer‐victimisation, but not physical peer‐victimisation. EEG evidence pointed to blunting of the brain response to social acceptance, as measured by the grand‐averaged ReWP event‐related potential (ERP) in adolescents and emerging adults, that was related to the degree of early peer victimisation (Rappaport et al., 2019). These authors found that blunting of the response to monetary rewards was not evident. Estimation of the location of the ReWP source points to the generation of this ERP component from the VS and the mPFC. In contrast, in an older sample of emerging mostly female adults, recent *relational* peer‐victimisation was associated with a blunted brain response to monetary rewards (Ethridge et al., 2018). This study did not find a significant effect of past‐year *physical* peer‐victimisation on the ReWP and did not investigate the brain response to social reward. In sexual minority youths, reports of victimisation (fighting, bullying, and safety) did not moderate brain region activation to social or monetary reward, anticipation, and feedback (Eckstrand et al., 2019). However, even though their subjective reports of victimisation did not modulate their brain response, sexual minority youths were hypothesised to experience a higher general expected level of exposure to social stressors compared to heterosexual controls, and exhibited lower right mPFC, left AI, and TPJ activation, in response to social but not monetary reward, which predicted severity of interpersonal depressive symptoms. In a younger female adolescent sample, peer‐victimisation during early adolescence was found to be associated with decreased response to potential monetary rewards in the mPFC, but no differences were found in the striatum or amygdala, nor did the study investigate brain response to social reward (Casement et al., 2014).

However, in the presence of peer‐evaluation or need for belonging to a social group, it appears that there is heightened increased evaluation of appetitive social stimuli. While in general, victimisation may be associated with blunting of brain response to social reward, this effect appears to be dependent on group‐inclusion status (in‐group or out‐group peers). Telzer and colleagues (Telzer et al., 2020) found that female adolescents with a history of greater exposure to peer‐victimisation displayed heightened activation in the amygdala, VS, fusiform gyrus, and TPJ, to in‐group relative to out‐group peers, suggesting greater anticipatory reward and outcome value to in‐group peers (i.e., peer‐group they are included in). In addition, peer‐victimisation also appears to affect the processing of social reward outcomes, which is associated with greater social anxiety. In a sample of early adolescents who were categorised as high‐ or low‐victimised, high victimisation was associated with greater right amygdala response to unpredictably positive peer evaluation, high wariness, and more severe social anxiety symptoms (Jarcho et al., 2019).

Structural differences associated with peer‐victimisation have been found in the striatal structures, both in the ventral (NAcc, part of the putamen ventral to the rostral internal capsule) and dorsal striatum (putamen). In a community sample of adolescents, peer‐victimisation was indirectly associated with generalized anxiety symptoms through decreases in putamen volumes. Individuals who were chronically victimised and displayed larger putamen volume at age 14, demonstrated steeper decreases in putamen volume compared to their less victimized counterparts, such that no significant difference in putamen volume was observed at age 19 (Quinlan et al., 2018). This finding was interpreted as differences in behavioural control and reward processing. Increased NAcc volume in female adolescents with major depressive disorder (MDD) when compared to healthy controls (Lee et al., 2020). In addition, NAcc volume was found to mediate the relationship between peer problems, measured as a combination of peer bullying‐perpetration and victimisation, and depression. The authors interpreted these differences in NAcc volume as demonstrating an increased sensitivity to social threat processing (a maintaining factor of social anxiety).

##### Social pain

Previous studies have demonstrated that exclusion during laboratory manipulations triggers activation of the same neural circuitry that underlies physical pain, including the dorsal anterior cingulate cortex (dACC), the subgenual anterior cingulate cortex (sgACC) and the anterior insula (AI) (Eisenberger, 2012; Eisenberger et al., 2003). Neural correlates of social exclusion are replicated in included study samples, across individuals with and without peer‐victimisation exposure. There consistent evidence from this review that increased intensity in the experience of social pain in peer‐victimised individuals during social exclusion, as a result of increased activation in the dACC, sgACC, and AI (Rudolph et al., 2016), as well as bilateral IFG and right occipital gyrus (Oppenheimer et al., 2020) and decreased functional connectivity between the left amygdala‐ACC and left amygdala‐right insula suggesting greater negative affective response during exclusion (McIver et al., 2019). Children who were chronically rejected by their peers exhibited greater dACC brain activity, during exclusion than on inclusion during the Cyberball paradigm, which is also consistent with greater rejection sensitivity (Will, van Lier et al., 2016).

Female adolescents with a history of chronic peer‐victimisation (relational, overt, and cyber) had increased sensitivity to social exclusion, characterised by heightened activation of brain regions implicated in the experience of social pain (dACC, sgACC, and AI) compared to those without history of peer‐victimisation (Rudolph et al., 2016). The activation of the social pain regions was associated with greater internalising symptoms in peer‐victimised female adolescents, but not female adolescents without a history of being peer‐victimised. In a sample of clinically anxious adolescents, peer‐victimisation and daily reported negative social experience (using ecological momentary assessment) was associated with increased activation in the right AI, bilateral IFG and right occipital gyrus, to social rejection, suggesting more intense experience of emotional pain (Oppenheimer et al., 2020). In adolescents with a history of peer‐victimisation, decreased functional connectivity between the left amygdala‐ACC and left amygdala‐right insula was observed during across both social inclusion and exclusion interaction with unfamiliar peers, suggesting greater negative affective response and greater anticipatory stress during social interactions with peers (McIver et al., 2019).

The studies examining neural responses to social exclusion have also found heightened activity in brain regions involved in emotion processing, including the amygdala, dorsolateral ACC, inferior fusiform gyrus, in peer‐victimised individuals compared to their non‐peer‐victimised peers (McIver et al., 2019, 2018; Rudolph et al., 2016; Will, Crone et al., 2016). McIver et al. (2018) found that individuals with high self‐reported bullying and victimisation showed increased neural response in the left amygdala, left parahippocampal gyrus, left inferior frontal operculum, and right fusiform gyrus. In particular, activation in the left parahippocampal gyrus was observed during social exclusion, which was deactivated in the control group, suggesting greater rejection sensitivity. In another study involving both male and female healthy adolescents, participants with a history of peer‐victimisation exhibited greater activation of brain regions not typically involved in processing social exclusion (i.e., social pain), but a pattern of more diffuse brain activation, including the amygdala, left parahippocampal gyrus (PHG), IFG and fusiform gyrus. The degree of activation in these areas was associated with individual severity of peer‐victimisation (McIver et al., 2019). Additionally, Will et al. (2016) found greater dACC brain activity during exclusion than on inclusion during the Cyberball paradigm in children who were chronically rejected by their peers. This finding is also consistent with the experience of greater rejection sensitivity reported by Rudolph et al. (2016).

#### Emotion processing and regulation

##### Emotion processing

In addition to heightened rejection sensitivity and experiencing of social pain, reduced medial OFC volume (Vargas et al., 2019) and fusiform gyrus increased fusiform thickness have been reported (Muetzel et al., 2019) in peer‐victimised older children and adolescents. There is mixed evidence as to whether peer‐victimised individuals show structural differences in the volume and GMV of the amygdala (Zhu, Zhou et al., 2019). No differences were found in the hippocampal volume (Lee et al., 2020; Muetzel et al., 2019; Vargas et al., 2019) nor white matter integrity of the uncinate fasciculus (Vargas et al., 2019). In terms of functional brain differences, there were contradictory reports of activation of the amygdala when presented with emotional faces (Baird et al., 2010; Swartz et al., 2020; Zhu, Zhou et al., 2019). Increased activation of other regions reported in victimised youths included the vmPFC (Weissman et al., 2019), left parahippocampal gyrus (PHG), IFG, and fusiform gyrus (McIver et al., 2019). Increased activation of the OFC, vlPFC and amygdala to negative performance feedback was also reported in victimised children (Lee et al., 2014).

***Structural Imaging Findings***. The studies included in this review report inconsistent findings; peer‐victimisation affects brain structures involved in emotion processing, such as the amygdala, in a developmentally sensitive manner. There is some evidence that early exposure results in an increase in amygdala volume while later exposure results in a loss of volume. In healthy young adults (aged 20–25), increased bilateral amygdala response to emotional faces versus shapes was associated with retrospective self‐report of peer emotional abuse during adolescence between the ages 13–15. In contrast, decreased amygdala response to emotional faces, compared to shapes, alongside reduced amygdala grey matter volume (GMV), was associated with peer physical abuse in early childhood, at ages 6 and 11 (Zhu, Lowen et al., 2019). However, in a large study involving children, no significant differences in amygdala or hippocampal volume were found in those classified as targets of frequent bullying (as gathered from teacher and parent report of physical, verbal, and relational peer‐victimisation; Muetzel et al., 2019). Similarly, in adolescent girls with MDD and healthy controls, peer‐victimisation was also not associated with volumetric differences in the amygdala and hippocampus (Lee et al., 2020). In another sample consisting of both healthy and clinically high‐risk adolescents, no significant associations were found between parent‐reported bullying, and the white matter integrity of the uncinate fasciculus, GMV of the amygdala or hippocampal regions (Vargas et al., 2019). However, reduced medial OFC volume, a region involved in both reward processing and emotion processing, was associated with parent‐reported bullying. Unfortunately, in this study, the developmental timing of exposure was not collected. Increased thickness of the fusiform gyrus has been found in 10 year‐old children classified as frequent targets of physical, relational and verbal bullying (Muetzel et al., 2019).

***Functional Imaging Findings.*** Inconsistent findings were also reported in amygdala activity in victimised adolescents. Amygdala activity in response to angry and fearful faces predicted *lower* self‐reported relational bullying and victimization in female adolescents (Baird et al., 2010). This finding was replicated in a sample of male and female healthy adolescents; higher bilateral amygdala activity in response to angry and fearful faces predicted high levels of peer‐victimisation measured by self‐report of peer aggression (Swartz et al., 2020). This heightened activation in the amygdala was found to be associated with further peer‐rejection/victimisation through increased social avoidance. However, increased activation in the amygdala was not found in female adolescents in association with self‐reported experience of relational peer aggression (Baird et al., 2010). An early study with a small sample of school‐aged children also found that those reporting high levels of victimisation, compared to controls, demonstrated greater activation in the amygdala (in addition to OFC/vmPFC, and vlPFC) when receiving negative feedback on their performance on an unsolvable puzzle (Lee et al., 2014). However, given that the manipulation in this experiment involved engaging in a demanding cognitive task in addition to processing the emotional valence of facial expressions, in the context of receiving interpersonal feedback, the diffuse pattern of brain regions activated may reflect the nature of the study design and choice of experimental paradigm (solving unsolvable geometric puzzles) rather than altered patterns of emotion processing.

In addition, a separate study combining fMRI during emotion face viewing with physiological measures of respiratory sinus arrhythmia (RSA) and skin conductance responses, found that exposure to threats in peer contexts during adolescence increased risk for later internalizing problems. This effect was found to be mediated by vmPFC‐RSA correlation (Weissman et al., 2019). Exposure to environmental threats enhanced the negative vmPFC–RSA correlation, although negative vmPFC–RSA correlation was associated with lower levels of internalizing problems, accounting for prior threat exposure. This suggests that the vmPFC regulation of the RSA is perhaps an adaptation to environmental threats, including bullying, with the enhancement of vmPFC‐RSA correlation compensating for increased emotional processing. As such, this may be a mechanism for implicit emotional regulation during emotional processing in individuals presenting with greater reactivity to emotionally relevant stimuli.

Finally, in the only study investigating the impact of chronic experience of cyberbullying in young adults aged 18–25, there were no significant differences between cyberbullied and non‐cyberbullied individuals in brain activation in areas including the left and right middle temporal gyrus, default mode network hubs, left and right posterior cerebellum/vermis, and putamen (McLoughlin et al., 2020). However, cyberbullied individuals demonstrated significantly lower activation of the precuneus, a hub within the default mode network, which is implicated in self‐evaluation and self‐consciousness. Due to this being small pilot study, the implications of these preliminary findings are unclear and beyond the scope of this review.

##### Emotion regulation

Both structural and functional differences in the vlPFC, dlPFC and OFC, implicated in goal‐directed regulation of emotion, have been reported in the included studies. Peer‐victimisation appears to be associated with these brain structures implicated in goal‐directed regulation of emotion. In boys with daily cortisol output, high victimization was associated with a smaller right vlPFC surface area; whereas in boys with a high daily cortisol output, high victimization was associated with a larger right vlPFC surface area (du Plessis et al., 2019). In addition, differences in vlPFC thickness dependent on cortisol level were also reported. fMRI evidence also found that peer‐rejected children compared to non‐rejected controls exhibited heightened neural responses, in the OFC, and vlPFC (in addition to the amygdala) when viewing video clips of facial expressions communicating negative feedback, suggesting both heightened sensitivity to negative emotions, and increased top‐down reappraisal (Lee et al., 2014). In adolescent girls, higher levels of peer‐victimisation predicted increased amygdala‐right vlPFC connectivity, suggesting less effective top‐down regulation of emotion in girls with high rejection sensitivity (Rudolph et al., 2021).

However, the overall recruitment of the dlPFC and vlPFC in victimised youths appears greater than in their non‐victimised peers. For example, Telzer et al. (2018) found that chronically peer‐victimised adolescents showed heightened activation in the vlPFC and dlPFC when making safe decisions during a risk‐taking driving simulation task. Neural patterns of brain activation in the dorsal striatum, and lateral PFC also revealed more difficulty in engaging with prosocial behaviours in victimised adolescents (Will, Crone et al., 2016). Chronically rejected adolescents, following experience of chronic exclusion in CyberBall, demonstrated increased dorsal striatum and lateral PFC activity when forgiving excluders (sharing money in a Dictator Game) than adolescents who were stably accepted by peers. Behaviourally, the rate of punishment and forgiveness of excluders did not differ between chronically rejected and stably accepted groups, but on a neural level, chronically rejected adolescents appear to have to exert greater cognitive control to achieve prosocial behavioural outcomes. A recent study including male and female adolescents in the community, self‐reports of relational, overt, and cyber peer‐victimisation were associated with increased VS‐IFG and VS‐vmPFC functional connectivity when perceiving high relative to low relational value during a social evaluation task (Fowler et al., 2021). The heightened connectivity between VS‐IFG may reflect an inability of the IFG to downregulate the VS in response to socially appetitive stimuli, reflecting the abnormally high perceived relational value of positive peer feedback in victimised peers, potentially explaining the tendency to seek social interactions despite victimisation and rejection.

#### Risk‐taking and social cognition

There is some in consistent findings on social cognition in association with peer‐victimisation. Telzer et al. (2018) found that chronically‐victimised female adolescents showed greater risk taking than non‐victimised female adolescents, but only following experience of exclusion. Compared to non‐victimised peers, the chronically rejected female adolescents also showed greater activation in brain areas involved in affective sensitivity (bilateral amygdala, VS, OFC), social cognition (TPJ, posterior superior temporal sulcus [pSTS], medial prefrontal cortex [MPFC], medial posterior parietal cortex [MPPC]), and cognitive control (dmPFC), when making risky decisions. This pattern of increased activation mediated self‐reported antisocial behaviours. Similarly, in female adolescents exposed to chronic victimisation, greater exposure to peer‐victimization was associated with heightened activation to in‐group relative to out‐group peers in the amygdala, VS, fusiform gyrus, and TPJ, as well as heightened social monitoring, which together would facilitate the drive for gaining (and regaining) social inclusion and connection (Telzer et al., 2020). The activation of social cognition regions (e.g., TPJ) appears to be associated with increased social monitoring and allocation of attention to in‐group peers (preferred social group), and not necessarily the ability to achieve better social outcomes or use of social skills, as the degree of activation in these areas was associated with greater internalising and externalising symptoms (Telzer et al., 2020). However, victimisation was not found to be significantly associated with brain activation in the right TPJ in sexual minority youths during social evaluation task (Eckstrand et al., 2019).

## DISCUSSION

The aim of this review was to address the questions whether (1) structural and functional brain differences are associated with peer‐victimisation, (2) whether structural and functional brain differences are associated with cyberbullying, (3) do structural and functional brain differences differ between traditional bullying and cyberbullying. We found that the current evidence was sufficient to fully address only the first question highlighted the need for further research to examine whether structural and functional brain differences were associated with cyberbullying, and whether structural and functional brain differences differed between traditional bullying and cyberbullying.

In this review of neuroimaging studies investigating the structural and functional brain differences associated with peer‐victimisation bullying and cyberbullying, we found evidence for behavioural and neurobiological differences between victimised and non‐victimised individuals, interacting with social, environmental and developmental factors, across a range of processes including peer‐evaluation, reward processing, social pain processing, emotion processing and regulation, as well as those for social cognition and risk taking. Peer‐victimisation has detrimental effects across various brain processes underlying peer‐evaluation (peer inclusion, rejection sensitivity, retaliation and forgiveness), is a risk factor for internalising symptoms (Rudolph et al., 2016), depression (Lee et al., 2015), anxiety in clinically anxious adolescents (Jarcho et al., 2019), and the positive symptom of psychosis (Vargas et al., 2019), as well as serving as a mediator between other types of maltreatment and depression (Zhu, Lowen et al., 2019). These reflect underlying individual differences and differential susceptibility to the impact of chronic and repetitive peer abuse.

There is some evidence to suggest peer‐victimisation is associated with reduced brain activation to reward stimulus, particularly in the ReWP ERP (Rappaport et al., 2019) and in the mPFC regions (Casement et al., 2014; Ethridge et al., 2018) in adolescents. In the presence of peer inclusion and peer‐evaluation, however, increased activity in the amygdala (Jarcho et al., 2019; Telzer et al., 2020), and VS (Telzer et al., 2020) was observed. There is clear evidence of increased intensity in the experience of social pain and rejection sensitivity in peer‐victimised individuals during social exclusion, as a result of increased activation in the dACC (Rudolph et al., 2016; Will, van Lier et al., 2016), sgACC, and AI (Rudolph et al., 2016), IFG (Oppenheimer et al., 2020) and right occipital gyrus (Oppenheimer et al., 2020) and decreased functional connectivity between the left amygdala‐ACC and left amygdala‐right insula (McIver et al., 2019) as well as between VS‐IFG and VS‐vmPFC (Fowler et al., 2021). The studies consistently showed that recruitment of the dlPFC and vlPFC in victimised youths appears greater than in their non‐victimised peers (Lee et al., 2014; Telzer et al., 2018, 2020; Will, Crone et al., 2016). The heightened response in these top‐down regulatory brain regions may reflect an overall tendency of recruitment required to exert cognitive control, for compensating the increased emotional reactivity or rejection sensitivity during experience of negative affect to social evaluation and social exclusion.

Additionally, a pattern of neural differences associated with peer‐victimisation exposure can be observed through the activation of neural regions involved in social cognition which requires the integration of cognition, emotion and motivation resulting in processing of social stimuli to direct social behaviour (Adolphs, 2001) during social evaluation as well as risk‐taking tasks. Increased activation of the MPFC, TPJ, pSTS, and MPPC suggesting social monitoring and allocation of attention to peers (Telzer et al., 2018, 2020). In addition, there is some evidence for other widespread brain during affect recognition and processing, particularly the fusiform gyrus (Lee et al., 2014; McIver et al., 2018), suggesting differences in facial processing associated with peer‐victimisation. Hence, in addition to the altered regulation of appetitive stimuli, risk‐taking in peer‐victimised adolescents appears to be driven by social cognition, such that heightened affective and reward sensitivity (increased perceived value of social reward and outcome), combined with increased concern for peer‐evaluation, contribute to heightened risk‐taking behaviours in peer‐victimised adolescents.

In terms of structural brain imaging, there is evidence for differences the vlPFC surface area (du Plessis et al., 2019), the striatal regions including the NAcc (Lee et al., 2020), putamen, and to a smaller extent the caudate (Quinlan et al., 2018), and OFC (vmPFC; Vargas et al., 2019). There is mixed evidence as to whether peer‐victimised individuals show structural differences in the volume and GMV of the amygdala, although there is some evidence that early exposure results in an increase in amygdala volume while later exposure results in a loss of volume (Zhu, Zhou et al., 2019). No differences associated with peer‐victimisation were found in the hippocampal volume (Lee et al., 2020; Muetzel et al., 2019; Vargas et al., 2019) nor white matter integrity of the uncinate fasciculus (Vargas et al., 2019).

One of the major areas of contradiction in this study was with the age‐related findings, both in terms of timing of peer victimisation (e.g., pre‐ vs. post‐pubertal) and the age range of study samples. There was a strong emphasis on investigating the impact of bullying on mental health outcomes in influencing the ongoing developmental processes in the brain, using samples including at least a proportion of children/adolescents, except for one paper (Ethridge et al., 2018). Differential effects of the developmental stage at which individuals experienced victimisation, for example, between childhood and adolescence, were explored (e.g., Zhu, Lowen et al., 2019). There were contradictory findings on the patterns of activation in the amygdala during processing of rewards and affect that were associated with exposure to peer‐victimisation (Lee et al., 2020; Muetzel et al., 2019; Vargas et al., 2019; Zhu, Lowen et al., 2019). One potential area of future research would be to elucidate how exposure to peer‐victimisation may be associated with the developmental changes in limbic and cognitive circuitry involved in emotional processes, which may begin at the subcortico‐subcortical moving to subcortico‐cortical to cortico‐subcortical and finally to cortico‐cortical level (Casey et al., 2017; Heller et al., 2016), which may provide an explanation for the age‐dependent findings of peer‐victimisation.

The quality ratings of included studies were mostly fair (*n* = 15), and good (*n* = 11). More longitudinal studies with multiple measures of exposure prior to measuring outcomes would improve the quality ratings and potentially help to untangle effects of pre‐existing predisposition that could have already been present before the emergence of a rejected peer status, compared to that as a result of chronic peer rejection, or even an interaction between both. In addition, a number of studies chose to investigate only one gender, potentially as a way of controlling for gender as a confounder while increasing the chances of observing the desired experimental effect of peer‐victimisation on developmentally sensitive processes, particularly in social cognition (Rudolph et al., 2016, 2021; Telzer et al., 2018), or biological sensitivity to stress (du Plessis et al., 2019). Larger sample sizes would increase the power to for comparisons between genders, age groups, and other individual characteristics, to identify differential neurobiological/psychological susceptibility to exposure of bullying and subtypes of peer abuse including relational‐peer victimisation, chronic rejection, and cyberbullying.

## CONCLUSION

This review has highlighted the recent progress in the application of a range of neuroimaging methods to investigating the effects of peer‐victimisation on neural processes contributing to psychopathological outcomes of children and adolescents, given that almost all the studies included (23 of 26) have been published in the last 5 years. However, it has also revealed many of the gaps in our current understanding, particularly with timing of exposure in relation to developmental age or puberty, and type of bullying victimisation exposure—whether this was relational, physical, or cyber. The findings point to patterns of both neural adaptation and sensitivity as a result of peer‐victimisation and bullying. This provides valued knowledge in informing the need for potential transdiagnostic interventions, targeting, or tailoring for highly victimised children and adolescents, such as addressing emotional dysregulation, rejection sensitivity, anhedonia, valuation of peers, and goal‐directed behaviours in regaining inclusion following exclusion/loss of status and risk‐taking behaviours. Finally, further studies in cyberbullying are warranted to help us draw any comparisons or conclusions as to whether its impact on brain structure and processes in childhood and adolescence is unique to this subtype of bullying.

## CONFLICTS OF INTEREST

Edward Barker is a member of the editorial advisory board for JCPP *Advances*. The remaining authors have declared that they have no competing or potential conflicts of interest.

## ETHICAL CONSIDERATIONS

This research was conducted in accordance with the principles embodied in the Declaration of Helsinki and in accordance with local statutory requirements. All the studies included in this review have been approved by their respective institutional or national research ethics committees and review boards.

## AUTHOR CONTRIBUTIONS

Conceived and designed the analysis (Tianyuan Ke, Edward Barker, Patrick Smith), collected the data (Tianyuan Ke, Sara De Simoni), contributed data or analysis tools (Tianyuan Ke, Sara De Simoni), performed the analysis (Tianyuan Ke, Sara De Simoni), wrote the paper (Tianyuan Ke), and other contribution (Sara De Simoni, Edward Barker, Patrick Smith).

## Supporting information

Supporting Information

## Data Availability

The authors confirm that the data supporting the findings of this study are available within the article and its supporting information. Requests for any further information can be submitted to the corresponding author, TK.
