# Supplementary material for: The association between peer‐victimisation and structural and functional brain outcomes: A systematic review
Source: JCPP Adv. 2022 May 12;2(2):e12081. doi: 10.1002/jcv2.12081 (PMC10242938; doi:10.1002/jcv2.12081)
Supplement: Supplementary file 1 — Supporting Information [file JCV2-2-e12081-s001.docx]

# Appendix S1

Search Strategy

**Search Strategy – Pubmed**

1)"bullying"[MeSH Terms] OR "bullying"[All Fields] OR "bullied"[All Fields] OR "bullies"[All Fields] OR "bully"[All Fields]

2) “peer victim*” "peer"[All Fields] AND (("victimisation"[All Fields] OR "victimisations"[All Fields]) OR "victimised"[All Fields])

3) “peer violence” or “peer intimidation” or “peer aggression”

4) "cyberbullied"[All Fields] OR "cyberbullies"[All Fields] OR "cyberbully"[All Fields] OR "cyberbullying"[MeSH Terms] OR "cyberbullying"[All Fields] OR “cybervictimi*ation” OR “cyber-victimi*ation”

5) “online bully*” OR “online bullies”

6) 1 or 2 or 3 or 4 or n5

7) "neural response"[All Fields] OR "neurobiologies"[All Fields] OR "neurobiology"[MeSH Terms] OR "neurobiology"[All Fields] OR "neurophysiology"[MeSH Terms] OR "neurophysiology"[All Fields]

8) "neuroimage"[All Fields] OR "neuroimaged"[All Fields] OR "neuroimagers"[All Fields] OR "neuroimages"[All Fields] OR "neuroimaging"[MeSH Terms] OR "neuroimaging"[All Fields] OR "neuroimagings"[All Fields]

9) “Functional neuroimaging”[MeSH]

10) “Brain mapping”[MeSH]

11) “connectome”[MeSH]

12) “(("magnetic resonance imaging"[MeSH Terms] OR (("magnetic"[All Fields] AND "resonance"[All Fields]) AND "imaging"[All Fields])) OR "magnetic resonance imaging"[All Fields]) OR "mri"[All Fields] OR “fMRI”[All Fields] OR “DTI” [All Fields]

13) “Electroencephalography”[MeSH] OR "electroencephalography"[All Fields] OR "eeg"[All Fields] OR "erp"

14) "magnetoencephalography"[MeSH Terms] OR "magnetoencephalography"[All Fields] OR “MEG”

15) “Brain waves”[MeSH]

16) “Electrocorticography”[MeSH]

17) “Electroencephalography phase synchronization” [MeSH]

18) (("evoked potentials"[MeSH Terms] OR ("evoked"[All Fields] AND "potentials"[All Fields])) OR "evoked potentials"[All Fields]) OR "erp"[All Fields]

19) “Cortical excitability” [MeSH]

20) 7 or 8 or 9 or 10 or 11 or 12 or 13 or 14 or 15 or 16 or 17 or 18 or 19

21) 6 AND 20

**Search Strategy – Psychinfo**

1. Bullying.mp. or exp bullying/ or relational aggression/ or school violence/ or teasing/ or bullying OR bullies OR bully OR peer victim* OR peer victimi*ation OR peer violence OR peer intimidation OR peer aggression
2. Cyberbullying.mp. or exp cyberbullying/ OR cyberbullied OR cyberbullies OR cyberbully* OR cybervictimi*ation OR cyber-victimi*ation OR online bully* OR online bullies
3. neuroimaging.mp. or exp neuroimaging/ or magnetic resonance imaging/ or action potentials/ or brain/ or brain connectivity/ or brain disorders/ or diffusion tensor imaging/ or evoked potentials/ or neuroimaging or magnetic resonance imaging or brain connectivity diffusion tensor imaging or evoked potentials
4. functional neuroimaging or brain mapping or connectome or magnetic resonance imaging or MRI or fMRI or DTI
5. electroencephalography/ or electrophysiology/ or alpha rhythm/ or beta rhythm/ or delta rhythm/ or gamma rhythm/ or theta rhythm/ or electrical activity/ or magnetoencephalography/ or mismatch negativity/ or neurobiological measures/ or p300/ Electroencephalography or electrophysiology or electrical activity or magnetoencephalography or electrophysiology
6. EEG or MEG or event-related potential or ERP
7. neurobiology.mp. or exp Neurobiology/ or neural response or neurophysiology.mp. or neurophysiology/
8. 1 OR 2
9. 3 or 4 or 5 or 6 or 7
10. 8 and 9

**Search Strategy – Psychinfo**

( KEY ( "bullying" ) OR TITLE-ABS-KEY ( "peer victim*" ) OR TITLE-ABS-KEY ( "peer violence" ) OR TITLE-ABS-KEY ( "peer intimidation" OR "relational aggression" OR "school violence" OR "teasing" OR " relational victimi*ation" OR "bullies" OR "bully" ) OR TITLE-ABS-KEY ( "peer aggression" ) OR ALL ( cyberbullying ) OR ALL ( "cybervictimi*ation" OR "cyber-victimi*ation" ) OR ALL ( "online bully*" OR "online bullies" ) ) AND ( ( TITLE-ABS-KEY ( neural AND response ) ) OR ( TITLE-ABS-KEY ( "neurobiolog*" OR "neurophysiolog*" OR "neuroimaging" OR "Functional neuroimaging" OR "Brain mapping" OR "connectome" OR "magnetic resonance imaging" OR "mri" OR "fmri" OR "diffusion tensor imaging" OR "dti" OR "Electroencephalography" OR "EEG" OR "Magnetoencephalography" OR "MEG" OR "Brain waves" OR "brain connectivity" OR "Evoked Potentials" OR "event-related potentials" OR "ERP" OR "Cortical excitability" ) ) ) AND ( LIMIT-TO ( PUBYEAR , 2020 ) OR LIMIT-TO ( PUBYEAR , 2019 ) OR LIMIT-TO ( PUBYEAR , 2018 ) OR LIMIT-TO ( PUBYEAR , 2017 ) OR LIMIT-TO ( PUBYEAR , 2016 ) OR LIMIT-TO ( PUBYEAR , 2015 ) OR LIMIT-TO ( PUBYEAR , 2014 ) OR LIMIT-TO ( PUBYEAR , 2013 ) OR LIMIT-TO ( PUBYEAR , 2012 ) OR LIMIT-TO ( PUBYEAR , 2011 ) OR LIMIT-TO ( PUBYEAR , 2010 ) OR LIMIT-TO ( PUBYEAR , 2009 ) OR LIMIT-TO ( PUBYEAR , 2008 ) OR LIMIT-TO ( PUBYEAR , 2007 ) OR LIMIT-TO ( PUBYEAR , 2006 ) OR LIMIT-TO ( PUBYEAR , 2005 ) OR LIMIT-TO ( PUBYEAR , 2004 ) OR LIMIT-TO ( PUBYEAR , 2003 ) OR LIMIT-TO ( PUBYEAR , 2002 ) OR LIMIT-TO ( PUBYEAR , 2001 ) OR LIMIT-TO ( PUBYEAR , 2000 ) ) AND ( LIMIT-TO ( DOCTYPE , "ar”

# Appendix S2

Quality ratings of included papers.

| Author | Year | 1 | 2 | 3 | 4 | 5 | 6 | 7 | 8 | 9 | 10 | 11 | 12 | 13 | 14 | Total | Rating |
| --- | --- | --- | --- | --- | --- | --- | --- | --- | --- | --- | --- | --- | --- | --- | --- | --- | --- |
| Baird et al. | 2010 | Y | Y | Y | Y | N | N | NA | Y | Y | N | Y | N | NA | Y | 8 | Fair |
| Casement et al. | 2014 | Y | Y | Y | Y | N | Y | Y | Y | Y | N | Y | N | Y | Y | 11 | Good |
| du Plessis et al. | 2019 | Y | Y | Y | Y | N | Y | N | Y | Y | Y | N | Y | Y | Y | 11 | Good |
| Eckstrand et al. | 2019 | Y | Y | Y | Y | Y | N | NA | Y | Y | N | Y | N | NA | Y | 9 | Fair |
| Ethridge et al. | 2018 | Y | Y | Y | Y | N | N | NA | Y | Y | N | Y | N | NA | Y | 8 | Fair |
| Fowler et al. | 2021 | Y | Y | Y | Y | N | N | NA | Y | Y | Y | N | N | NA | Y | 8 | Fair |
| Jarcho et al. | 2019 | Y | Y | Y | Y | Y | N | NA | N | Y | N | Y | N | NA | Y | 7 | Fair |
| Lee, H. S. et al., | 2014 | Y | Y | Y | Y | N | N | N | Y | N | N | Y | N | NA | Y | 7 | Fair |
| Lee, K. H. et al. | 2020 | Y | Y | CD | Y | N | N | N | Y | Y | N | Y | N | NA | Y | 7 | Fair |
| McIver et al. | 2019 | Y | Y | Y | Y | N | N | NA | Y | Y | N | Y | N | NA | Y | 8 | Fair |
| McIver et al. | 2018 | Y | Y | N | Y | N | N | NA | N | Y | N | Y | N | Y | Y | 7 | Fair |
| McLoughlin et al. | 2020 | Y | Y | Y | Y | N | N | NA | N | Y | N | Y | N | NA | Y | 7 | Fair |
| Muetzel et al. | 2019 | Y | Y | CD | Y | N | Y | Y | Y | Y | N | Y | N | NA | Y | 9 | Fair |
| Oppeheimeret al. | 2020 | Y | Y | Y | Y | N | N | NA | Y | N | Y | Y | N | Y | Y | 9 | Fair |
| Quinlan et al. | 2018 | Y | Y | Y | Y | Y | Y | Y | Y | Y | Y | Y | N | Y | Y | 13 | Good |
| Rappaport et al. | 2019 | Y | Y | NR | Y | Y | Y | Y | Y | Y | Y | Y | N | NR | Y | 11 | Good |
| Rudolph et al. | 2016 | Y | Y | Y | Y | N | Y | Y | N | Y | Y | Y | N | Y | Y | 11 | Good |
| Rudolph et al. | 2020 | Y | Y | CD | Y | N | Y | Y | Y | Y | Y | Y | N | Y | Y | 12 | Good |
| Swartz et al. | 2020 | Y | Y | Y | Y | N | N | ? | Y | Y | N | Y | N | NA | Y | 8 | Fair |
| Telzer et al. | 2019 | Y | Y | N | Y | N | Y | Y | Y | Y | Y | Y | N | Y | Y | 11 | Good |
| Telzer et al. | 2018 | Y | Y | N | Y | N | Y | Y | Y | Y | Y | Y | N | NA | Y | 11 | Good |
| Vargas et al. | 2019 | Y | Y | Y | Y | Y | N | NA | Y | Y | N | Y | N | NA | Y | 9 | Fair |
| Weissman et al. | 2019 | Y | Y | N | Y | N | Y | Y | Y | Y | Y | Y | N | Y | Y | 11 | Good |
| Will, van Lier, Crone & Guroglu | 2016 | Y | Y | N | Y | Y | Y | Y | N | N | Y | Y | N | Y | Y | 10 | Good |
| Will, Crone, Van Lier, Güroğlu, & Güroǧlu | 2016 | Y | Y | N | Y | Y | Y | Y | N | N | Y | Y | N | Y | Y | 10 | Good |
| Zhu et al. | 2019 | Y | Y | Y | Y | N | N | NA | N | Y | Y | Y | N | NA | Y | 8 | Fair |
| \|  \| Key \|  \| \| --- \| --- \| --- \| \| 1 \| Was the research question or objective in this paper clearly stated? \| \| \| 2 \| Was the study population clearly specified and defined? \| \| \| 3 \| Was the participation rate of eligible persons at least 50%? \|  \| \| 4 \| Were all the subjects selected or recruited from the same or similar populations (including same time period)? Were inclusion/exclusion criteria prespecified and applied uniformly to all participants? \| \| \| 5 \| Was a sample size justification, power description, or variance and effect estimates provided? \| \| \| 6 \| For the analyses, were the exposures of interest measured prior to the outcomes being measured? \| \| \| 7 \| Was the timeframe sufficient so that one could reasonably expect to see an association between exposure and outcome if it existed? \|  \| \| 8 \| For exposures that can vary in amount or level, did the study examine different levels of the exposure as related to outcome (e.g. categories of exposure/exposure measured as continuous variable)? \| \| \| 9 \| Were the exposure measures (IVs) clearly defined, valid, reliable, and implemented consistently across all study participants? \| \| \| 10 \| Was the exposure assessed more than once over time? \|  \| \| 11 \| Were the outcome measures (DVs) clearly defined, valid, reliable and implemented consistently across all study participants? \| \| \| 12 \| Were the outcome assessors blinded to the exposure status of participants? \| \| \| 13 \| Was loss to follow-up after baseline 20% or less? \| \| \| 14 \| Were key potential confounding variables measured and adjusted statistically for their impact on the relationship between exposure and outcome? \| \| \| CD: Cannot determine; NA: Not applicable; NR: Not reported; N: No; Y: Yes \| \| \| | | | | | | | | | | | | | | | | | |
